# *Candida* Non-*albicans* and Non-*auris* Causing Invasive Candidiasis in a Fourth-Level Hospital in Colombia: Epidemiology, Antifungal Susceptibility, and Genetic Diversity

**DOI:** 10.3390/jof10050326

**Published:** 2024-04-30

**Authors:** Juan Camilo Hernández-Pabón, Bryan Tabares, Óscar Gil, Carlos Lugo-Sánchez, Aldair Santana, Alfonso Barón, Carolina Firacative

**Affiliations:** 1 Group MICROS Research Incubator, School of Medicine and Health Sciences, Universidad de Rosario, Bogota 111221, Colombia; juancamilo.hernand04@urosario.edu.co (J.C.H.-P.); oscari.gil@urosario.edu.co (Ó.G.); carlosf.lugo@urosario.edu.co (C.L.-S.); 2 Unidad de Extensión Hospitalaria, Hospital Universitario Mayor Méderi, Bogota 111411, Colombia; bryan.tabares@urosario.edu.co; 3 Clinical Laboratory and Transfusion Service, Hospital Universitario Mayor Méderi, Bogota 111411, Colombia; aldairasc@gmail.com; 4 Department of Medical Clinics, Hospital Universitario Mayor Méderi, Bogota 111411, Colombia; baron_sanchez@hotmail.com; 5 Studies in Translational Microbiology and Emerging Diseases (MICROS) Research Group, School of Medicine and Health Sciences, Universidad de Rosario, Bogota 111221, Colombia

**Keywords:** antifungal drug resistance, *Candida*, Colombia, epidemiology, healthcare-associated infections, invasive fungal infections

## Abstract

Increasingly common and associated with healthcare settings, *Candida* infections are very important, since some species of this genus can develop antifungal resistance. We contribute data on the epidemiology, antifungal susceptibility, and genetic diversity of *Candida* non-*albicans* and non-*auris* affecting critically ill patients in a fourth-level hospital in Colombia. Ninety-seven isolates causing invasive infections, identified by conventional methods over 18 months, were studied. Data from patients affected by these yeasts, including sex, age, comorbidities, treatment, and outcome, were analysed. The antifungal susceptibility of the isolates was determined, and the ribosomal DNA was sequenced. *Candida parapsilosis*, *Candida tropicalis*, *Candida glabrata*, *Candida dubliniensis*, and *Candida guilliermondii* caused 48.5% of all cases of invasive candidiasis. The species were mainly recovered from blood (50%). Patients were mostly men (53.4%), between 18 days and 93 years old, hospitalized in the ICU (70.7%). Overall mortality was 46.6%, but patients in the ICU, using antibiotics, with diabetes mellitus, or with *C. glabrata* infections were more likely to die. Resistant isolates were identified in *C. parapsilosis*, *C. tropicalis*, and *C. glabrata*. This study provides epidemiological data for the surveillance of emerging *Candida* species, highlighting their clinical impact, as well as the emergence of antifungal resistance and clonal dispersal.

## 1. Introduction

Invasive candidiasis, comprising both candidemia and deep-seated infection, is the most frequent mycosis among hospitalized patients in the world [1,2]. With an estimated 750,000 cases occurring yearly, this mycosis has mortality rates that often exceed 40%, despite patients receiving antifungal therapy [3,4]. Candidemia, particularly, appears as the fourth leading cause of healthcare-associated bloodstream infections (BSIs) in intensive care units (ICUs), following the bacterial agents *Escherichia coli*, *Staphylococcus aureus*, *Klebsiella pneumoniae*, and *Streptococcus pneumoniae* [5]. Notably, about 80% of cases of candidemia occur in patients with iatrogenic or nosocomial factors, including the presence of central venous catheters (CVCs) and intravascular or intracranial devices, as well as the use of broad-spectrum antibiotics and recent abdominal surgery [6,7].

Depending on the country, local epidemiology, age of the patient, and other factors, the annual incidence of invasive candidiasis considerably differs, being significantly higher in the USA (10–24 cases per 100,000 inhabitants), and lower in Australia, Canada, Europe, and Latin America (1.4–10.4 cases per 100,000 inhabitants) [8]. Similarly, population-based epidemiological studies on candidemia have shown that the number of cases of this fungemia per 1000 admissions per year presents broad variation among countries [1,8]. In Colombia, an incidence of 1.96 episodes per 1000 hospital admissions has been estimated, which is much higher than rates reported in other Latin American countries [9,10].

Regarding aetiological agents, it is well known that the distribution and occurrence of *Candida* species causing infection has changed over recent decades and that the epidemiology of these infections continues to shift [11]. Globally, *Candida albicans* remains the leading agent not only of candidemia but also of other forms of invasive candidiasis, accounting for about half the number of cases [12,13]. However, the frequency of non-*albicans* species, including *Candida glabrata* (*Nakaseomyces glabratus*), *Candida parapsilosis*, *Candida tropicalis*, and *Candida krusei* (*Pichia kudriavzevii*), among others, is increasing with a significant variability in the relative frequency of each species within and between geographical regions and over time [14]. In addition, in many parts of the world, including Colombia, *Candida auris* has emerged and settled as a major multidrug-resistant pathogen in healthcare facilities [15,16].

The increasing incidence of *Candida* species other than *C. albicans* is of great concern, mainly when selecting empiric antifungal therapy, as some non-*albicans* species are intrinsically resistant or have acquired antifungal resistance to azoles and, to a lesser extent, to echinocandins [17]. Moreover, together with *C. albicans*, both *C. tropicalis* and *C. glabrata* have been described to be more virulent than *C. parapsilosis* and *C. krusei* in an immunocompetent animal model, which could be related to the variation in mortality rates among patients between species [18]. Given that *C. glabrata*, *C. tropicalis, C. parapsilosis*, and *C. krusei* are pathogens for which treatment and management challenges exist, these species were recently included by the World Health Organization (WHO) in the fungal priority pathogens list to focus attention on their public health importance [19].

Considering the ongoing rise in the prevalence of non-*albicans Candida* in the world and the preoccupying appearance of resistant isolates, it is important to obtain data on the local epidemiology and antifungal susceptibility of these pathogens affecting critically ill patients. Therefore, we aimed to provide a deeper insight into the epidemiological characteristics of patients with candidemia and other invasive infections caused by non-*albicans* and non-*auris* species in a fourth-level hospital in Bogota, Colombia, and to determine the antifungal susceptibility profiles of these yeasts. With this study, we contribute data to the surveillance of non-*albicans* and non-*auris Candida* species in Colombia and globally. Remarkably, the participating hospital is one of largest hospitals in the country, with a capacity of 965 beds and advanced surgical equipment and diagnostic techniques.

## 2. Materials and Methods

### 2.1. Patients and Isolates

Over a period of 18 months, from January 2022 to June 2023, patients with fungaemia and other invasive infections caused by *Candida* non-*albicans* and *Candida* non-*auris* were recorded by the Clinical Laboratory and Transfusion Service at Hospital Universitario Mayor Méderi in Bogota, Colombia, a fourth-level healthcare institution. Isolates were identified in the hospital by routinely used methods, including BD Phoenix™ (Becton, Dickinson and Company, Franklin Lakes, NJ, USA) and MALDI-TOF MALDI Biotyper^®^ (Bruker Daltonics Inc., Bremen, Germany). Data from the medical records of patients affected by these yeasts were tabulated in Microsoft Excel, including epidemiological data on sex, age, underlying conditions or risk factors, treatment for the mycosis and related diseases, length of stay in hospital, type of hospital bed (general wards or units), and outcome. The clinical sample from which the isolate of *Candida* was recovered, together with the species of *Candida* identified, was registered as well. Coinfection with other *Candida* species, as well as other fungi, bacteria, and/or viruses was considered too. For some patients more than one clinical sample was collected; as such, more than one isolate of *Candida* was recovered. *C. auris* isolates were not included in the present study considering that, since 2016, this *Candida* species has been subject to mandatory notification in the country and there is a specific surveillance program addressed to characterise the epidemiology of infections caused by this pathogen [20]. Nevertheless, the number of both *C. albicans* and *C. auris* infections was taken into account when estimating the prevalence of non-*albicans* and non-*auris* cases. Similarly, the species and number of bacteria causing bacteraemia in the studied period were taken into consideration to estimate the percentage of BSI caused by *Candida* species in the hospital.

After identification in the hospital, isolates were transported using Transystem™ 114C swabs (Copan, Murrieta, CA, USA), which are regular rayon swabs with amies agar gel and charcoal, to the MICROS Group laboratory at Universidad del Rosario to carry out further experiments. On some occasions, the species of certain isolates were identified in the hospital and reported; however, these isolates were not viable after transportation, mainly due to contamination with bacteria and moulds. As such, there is no data on all experiments for all isolates reported. In other cases, informed consent from some patients was not obtained; hence, the isolates recovered from them were not included in the epidemiological analysis.

### 2.2. Antifungal Susceptibility

Experiments to determine susceptibility to anidulafungin, micafungin, caspofungin, 5-fluorocytosine, posaconazole, voriconazole, itraconazole, fluconazole, and amphotericin B were carried out using the colorimetric broth microdilution test and Sensititre^®^ YeastOne^®^ plates (Thermo Scientific, Waltham, MA, USA), following the manufacturer’s instructions. Briefly, each isolate was grown on Sabouraud dextrose agar and incubated at 35 °C for 24 h. Afterwards, an inoculum was prepared per isolate in 5 mL of sterile water and adjusted to a density of 0.5 McFarland standard (1–5 × 10^6^ cells/mL). From each cell suspension, 20 µL was transferred into a tube containing 11 mL of YeastOne^®^ inoculum broth to obtain a final concentration of 1.8–9 × 10^3^ cells/mL. From the last suspension, 100 µL was placed into each well of each Sensititre^®^ YeastOne^®^ plate. Plates were sealed and incubated at 35 °C for 24 h. Colorimetric minimum inhibitory concentration (MIC) was defined as the lowest concentration of each antifungal that prevented the development of a pink or fuchsia colour (first blue well (no growth)) for amphotericin B, or the first purple well (growth inhibition) or blue well (no growth) for echinocandins, 5-fluorocytosine, and azoles [21]. *C. krusei* ATCC^®^ 6258 and *C. parapsilosis* ATCC^®^ 22019 were used as quality control strains as they have defined MIC ranges, according to the M27M44S guideline of the Clinical and Laboratory Standards Institute (CLSI) [22]. The purity of each cell suspension and colony counts were determined by plating 20 µL of the 1.8–9 × 10^3^ cells/mL inoculum on Sabouraud dextrose agar incubated at 35 °C for 24 h.

The ranges of drug concentrations, tested by 2-fold serial dilutions, were 0.015625–8 µg/mL for anidulafungin, 0.0078125–8 µg/mL for micafungin, caspofungin, voriconazole, and posaconazole, 0.015625–16 µg/mL for itraconazole, 0.125–256 µg/mL for fluconazole, 0.0625–64 µg/mL for 5-fluorocytosine, and 0.125–8 µg/mL for amphotericin B. For each species of *Candida*, the frequency of MIC values and the geometric mean MIC of each antifungal drug were determined. When available, clinical breakpoints per species and per drug, established by the CLSI, were consulted to determine if the isolates of this study were susceptible, susceptible dose-dependent (SDD), or resistant to certain antifungal drugs [22]. This was undertaken by considering the agreement between commercial tests, such as Sensitire^®^, YeastOne^®^, and the CLSI methodology [21].

### 2.3. DNA Extraction

Each isolate was cultured on Sabouraud dextrose agar and incubated at 35 °C for 24 h prior to genomic DNA extraction, which was performed as previously described [23] with some modifications. For each isolate, two to three colonies were placed separately in 1.5 mL tubes and kept at −20 °C overnight. Subsequently, 500 µL of lysis buffer (17.3 mM SDS, 0.25 M NaCl, 25 mM EDTA, 0.2 M Tris-HCl) and 5 µL of 2-mercaptoethanol were added, mixed vigorously by vortex, and incubated at 65 °C for 1 h. After incubation, 500 µL of phenol:chloroform:isoamyl alcohol (25:24:1) was added and mixed by vortex thoroughly for 2 min to obtain a homogeneous suspension. Next, the tubes were centrifuged at 14,000 rpm for 15 min. DNA from the aqueous phase was transferred to a new 1.5 mL tube and an equal amount of cold isopropanol was added to precipitate the DNA by incubation at −20 °C overnight. The precipitated DNA was pelleted by centrifugation at 14,000 rpm for 15 min and washed with 500 µL 70% cold ethanol. The resulting DNA pellet was dried at room temperature and diluted in sterile deionized water. DNA concentration was determined by a spectrophotometer and adjusted to 10 ng/µL.

### 2.4. Internal Transcribed Spacer (ITS) Amplification and Sequencing

To confirm the species identification of the isolates, amplification of the ITS region was performed according to the International Society for Human and Animal Mycology (ISHAM) barcoding scheme with the primers SR6R (AAGTARAAGTCGTAACAAGG) and LR1 (GGTTGGTTTCTTTTCCT) [24]. PCR products were commercially purified and sequenced, both forward and reverse strands, by GenCore at Universidad de los Andes, Bogota, Colombia. Sequences were edited and contigs were assembled using the program Sequencher 5.4.6 (Gene Codes Corporation, Ann Arbor, MI, USA). Assembled sequences of the studied isolates, together with ITS sequences from the reference strains of *C. albicans* CBS5983 (KY101838), *C. dubliniensis* CBS7987 (KY102055), *C. glabrata* CBS138 (KY102103), *C. parapsilosis* CBS604 (FJ872016), and *C. tropicalis* CBS1920 (MW284468), were aligned with the software Mega 11 [25]. In addition, reference strains of *Candida orthopsilosis* CBS 10906 (FJ872018) and *Candida metapsilosis* CBS 10907 (FJ872019) were included to rule out misidentification among the *C. parapsilosis* isolates [24,26]. Using Mega 11, a dendrogram showing the genetic relationship between isolates was constructed using maximum-likelihood analysis with a bootstrap analysis of 100 replicates and the Jukes–Cantor model [25]. To estimate the genetic diversity per species, the Simpson’s diversity index (D) was calculated with the number and frequency of haplotypes [27]. The intraspecies diversity was calculated with the nucleotide diversity, π, the haplotype (gene) diversity, Hd, and the number of segregating polymorphic sites (SSs) using the software DnaSP 6.12.03 [28].

### 2.5. Statistical Analyses

Epidemiological data are shown as numbers and percentages. For quantitative variables, means and ranges are shown. A multivariate logistic regression was used to assess the relation between outcome of patients and the explanatory variables of (i) sex, (ii) age, (iii) length of hospital stay, (iv) diagnosis, (v) hospitalization in general ward or ICU, and (vi) *Candida* species. In addition, a second multivariate logistic regression was performed to assess the relation between the species of *Candida* causing the infection and the variables of (i) sex, (ii) age, (iii) length of hospital stay, and (iv) diagnosis of patients. Data were checked for multicollinearity with the Belsley–Kuh–Welsch technique. Heteroskedasticity and the normality of residuals were assessed, respectively by the White test and the Shapiro–Wilk test. Adjusted odds ratios (OR) and 95% confidence intervals (CI) are presented. The association between outcome of patients and the explanatory variables of (i) risk factor or underlying condition, (ii) diagnosis, and (iii) *Candida* species was tested with the Chi-squared test. The alpha risk was set to 5% (α = 0.05).

Differences in MIC values between species of *Candida* were established per antifungal drug using the Mann–Whitney test. Statistical analysis was performed with the online platform EasyMedStat version 3.9 (Accessed from 29 November 2023 to 7 February 2024, www.easymedstat.com) and the software GraphPad Prism 9 (La Jolla, CA, USA). *p*-values < 0.05 were considered statistically significant.

## 3. Results

### 3.1. Candida Non-albicans and Non-auris Caused around Half the Number of Cases of Invasive Candidiasis with a Predominance of C. parapsilosis

**Figure 1:**
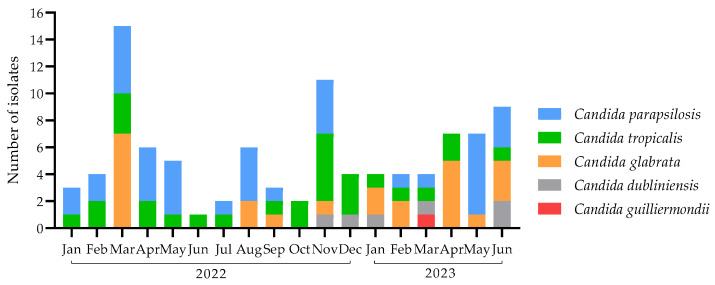
Distribution of *Candida* species per month during the studied period. In our hospital, 38 cases of *Candida parapsilosis*, 28 of *Candida tropicalis*, 24 of *Candida glabrata*, 6 of *Candida dubliniensis*, and 1 of *Candida guilliermondii* were reported over a period of 18 months.

During the 18 months of the studied period, 200 cases of invasive infection caused by *Candida* species were identified and reported in the hospital. From these, *C. albicans* was the predominant species, causing 74 cases (37%). From the remaining episodes of invasive candidiasis, *C. auris* caused 29 cases (14.5%), and other species, together, caused 97 cases (48.5%), representing almost half the number of total cases. From the non-*albicans* and non-*auris Candida*, five species were identified, predominantly *C. parapsilosis* with 38 cases (19%), followed by *C. tropicalis* with 28 cases (14%), *C. glabrata* with 24 cases (12%), *Candida dubliniensis* with 6 cases (3%), and *Candida guilliermondii* (*Meyerozyma guilliermondii*) with 1 case (0.5%). Through the studied period, *C. parapsilosis* and *C. tropicalis* were commonly recovered, while *C. glabrata* was reported only in some months, and *C. dubliniensis* seems to have emerged in November 2022, after which point it was recovered in the hospital (Figure 1).

Notably, from the total number of cases of invasive candidiasis caused by any *Candida* species, candidemia alone was reported in 175 cases (87.5%). Accounting for all cases of BSI diagnosed in the studied period in the hospital, candidemia occupied the fifth place (3.5%), after bacteraemia caused by the Gram-negative bacteria *E. coli* (27.6%) and *K. pneumoniae* (18.1%), and the Gram-positive bacteria *S. aureus* (14.3%) and *Staphylococcus epidermidis* (5.5%). In the ICU in particular, BSI caused by *Candida* species was in the fifth position as well; however, the frequency of the bacteria differed, as *K. pneumoniae* was the predominant aetiological agent (20.8%), followed by *S. aureus* (17.1%), *E. coli* (15.2%), and *S. epidermidis* (11.1%).

### 3.2. Clinical Characteristics of Patients Differ Depending on the Candida Species Causing Infection

**Figure 2:**
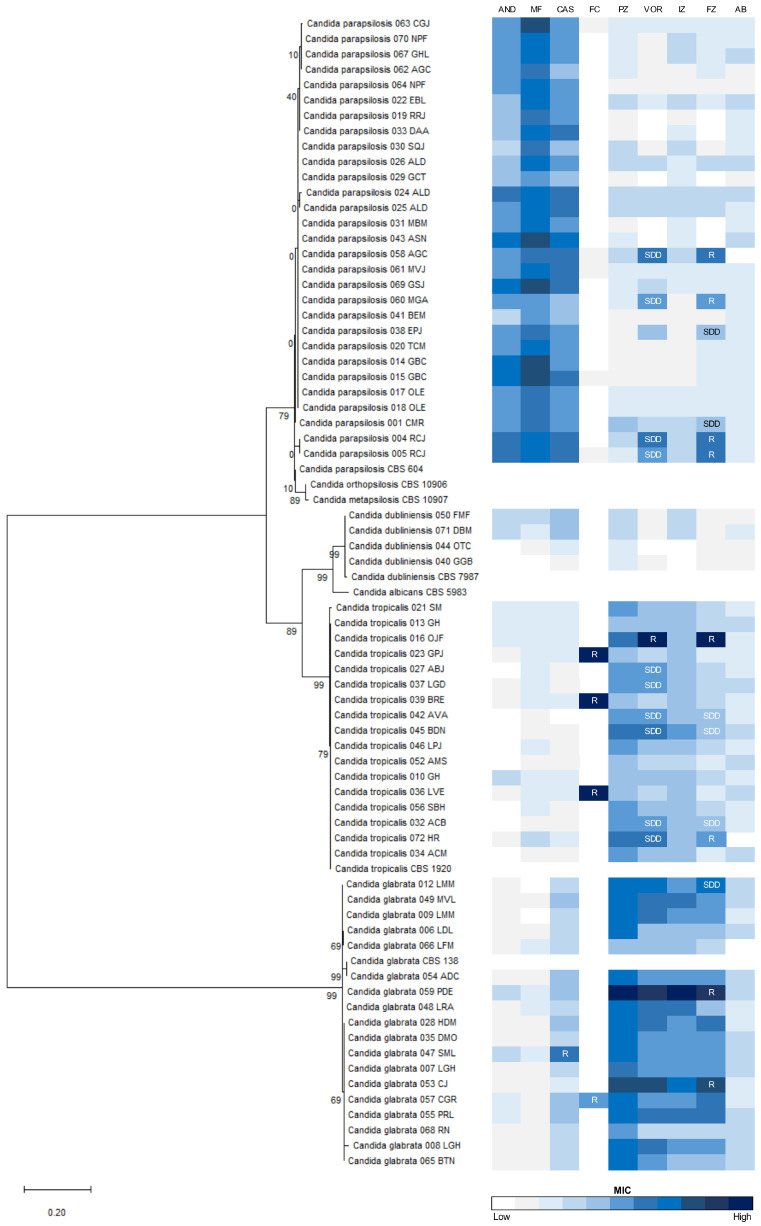
Dendrogram showing the genetic relationships, based on ribosomal DNA sequences, and the antifungal susceptibility of the *Candida* isolates in this study. Internal transcribed spacer (ITS) sequences from 68 *Candida* non-*albicans* and non-*auris* isolates, together with the reference strains of *Candida albicans* (CBS5983), *Candida dubliniensis* (CBS7987), *Candida glabrata* (CBS138), *Candida parapsilosis* (CBS604), *Candida orthopsilosis* (CBS 10906), *Candida metapsilosis* (CBS 10907), and *Candida tropicalis* (CBS1920), are shown. In front of each isolate, minimum inhibitory concentration (MIC) values to anidulafungin (AND), micafungin (MF), caspofungin (CAS), 5-fluorocytosine (FC), posaconazole (PZ), voriconazole (VOR), itraconazole (IZ), fluconazole (FZ), and amphotericin-B (AMB) are represented in the colour scale. Isolates that are resistant (R) or susceptible-dose-dependent (SDD) to certain antifungal drugs are indicated.

From the 97 cases of non-*albicans* and non-*auris Candida* identified in this study, 68 (72.3%) isolates from 58 patients were included in the epidemiological analyses. From these 68 isolates, whose identification was confirmed by the sequencing of ITSs, 29 (42.6%) were *C. parapsilosis*, 18 (26.5%) were *C. glabrata*, 17 (25%) were *C. tropicalis*, and 4 (5.9%) were *C. dubliniensis* (Figure 2). Unfortunately, the only isolate of *C. guilliermondii* reported in this study was contaminated with moulds during transportation; as such, this species was not included in further analysis. From 51 patients, a unique *Candida* isolate was recovered from a unique clinical sample. However, from three patients, two different blood samples were taken, and one unique isolate of *C. parapsilosis* was recovered from each blood sample. Similarly, from two patients, two different blood samples were taken and a unique isolate of *C. glabrata* was recovered from each blood sample. In one patient, three isolates of *C. parapsilosis* were recovered from three different blood samples. From two patients, two different isolates of *C. parapsilosis* were recovered from blood and catheter tip cultures. Co-infection with more than one species of *Candida* was not reported in any patient.

The demographic and clinical characteristics of the 58 studied patients are summarized in Table 1. Of the patients, 31 (53.4%) were men and 27 (46.6%) were women. Cases are described from an 18-day-old infant to a 93-year-old patient, with an average age of 63.1 years and a median of 67 years.

Among the 58 studied patients, candidemia alone was diagnosed in 29 cases (50%), followed by 21 cases (36.2%) of other invasive infections and 8 cases (13.8%) of urinary tract infection. Apart from blood and urine, *Candida* species were recovered from bronchoalveolar lavage (BAL), peritoneal lavage, and catheter tips, among other various secretions and clinical samples.

With respect to the etiological agent, patients with *C. parapsilosis* infections appeared to be younger (55.96 ± 23.01 years) than patients affected by *C. tropicalis* (67.65 ± 14.45) or *C. glabrata* (67.55 ± 15.18) (*p* < 0.01). Men were more likely to be infected by *C. parapsilosis* (OR:0.29; CI: 0.12–0.7, *p* = 0.009), while women were more likely to develop infection by *C. glabrata* (OR:5.77; CI: 1.94–17.17, *p* = 0.0016). *C. tropicalis* affected men and women equally (OR: 0.56; CI: 0.229–1.37, *p* = 0.203). *C. parapsilosis* was more likely to cause candidemia (OR: 5.1; CI: 2–13 with *p* < 0.001), while *C. tropicalis* was more likely to cause other forms of invasive candidiasis (OR: 0.368; CI: 0.149–0.907 with *p* = 0.0299). *C. glabrata* caused candidemia and other infections equally (OR:0.56; CI. 0.22–1.41, with *p* = 0.2183). Length of hospital stay did not differ between infections caused by different species.

Regarding underlying conditions or risk factors, the most commonly reported among patients were antibiotic use, bacterial co-infection, cancer, high blood pressure, antihypertensives use, and diabetes mellitus (Table 1). While some patients presented one to three underlying conditions or risk factors, most patients (74.1%) had four or more. Although *C. parapsilosis* has often been associated with CVC placement and *C. tropicalis* with patients with haematological malignancies [29], there was no association between the underlying conditions or risk factors of our patients and the species of *Candida* causing the infection.

**Figure 3:**
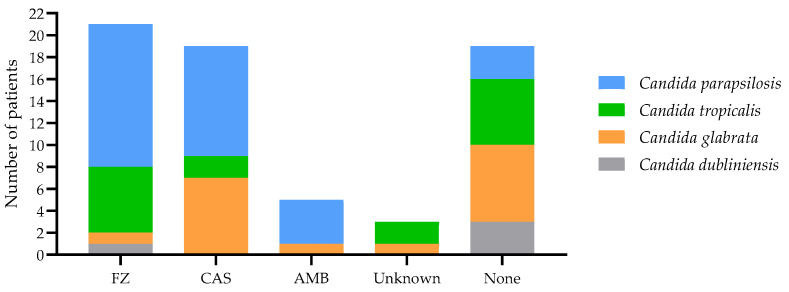
Distribution of *Candida* species according to the treatment. The treatment received by the patients from our hospital, according to the species of *Candida* causing the infection, is shown. AMB: amphotericin-B; CAS: caspofungin; FZ: fluconazole.

Most patients (46.5%) received monotherapy either with fluconazole (24.1%), caspofungin (19%), or amphotericin B (3.4%). In nine patients (15.5%), combined therapy, mainly fluconazole plus caspofungin, was given. Three (5.2%) patients received antifungal treatment, but this was not specified as the files of the patients were incomplete. Nineteen (32.8%) patients did not receive any antifungal treatment (Table 1). *C. parapsilosis* infections were mostly treated with fluconazole and caspofungin, while those patients affected by *C. tropicalis* were mostly treated with fluconazole or not treated. Similarly, most patients affected by *C. glabrata* were treated with caspofungin or not treated (Figure 3).

Of the patients, 31 (53.4%) survived and 27 (46.6%) died. Multivariate analysis revealed that sex, age, length of stay in hospital, and diagnosis were not associated with the outcome of patients. However, patients in the ICU were found to be more likely to die than patients hospitalized in the general ward (OR: 0.36; CI: 0.153–0.847; *p* < 0.05), and there was a statistically significant higher risk of dying of *C. glabrata* infection (OR: 0.304; CI: 0.104–0.894; *p* < 0.05) compared to infections caused by other *Candida* species (Table 2). Univariate analysis revealed that patients using antibiotics were statistically more likely to die than patients with other risk factors or underlying conditions, while patients with diabetes mellitus were more likely to survive (Table 3). Generally, infections caused by *C. glabrata* were found more likely to cause death, with a crude mortality rate of 62.5%.

### 3.3. Antifungal Susceptibility Profiles Vary among Candida Species

The majority of *Candida* isolates in this study were susceptible to the echinocandins tested, to fluconazole, and to voriconazole, according to the CLSI breakpoints [22] (Table S1). However, from the 29 *C. parapsilosis* isolates, 4 (13.8%) were concomitantly resistant to fluconazole and SDD to voriconazole. In addition, two additional *C. parapsilosis* isolates (6.9%) were SDD to fluconazole. Of the seventeen *C. tropicalis* isolates, one (5.9%) was simultaneously resistant to fluconazole and voriconazole, three (17.6%) were SDD to both azoles, one (5.9%) was resistant to fluconazole and SDD to voriconazole, and two (11.8%) were SDD to voriconazole. Even though the CLSI has not set the breakpoint for 5-fluorocytosine, three (17.6%) isolates of *C. tropicalis* had a high MIC to this antifungal (64 µg/mL), and as such were considered resistant. From the eighteen *C. glabrata* isolates, one (5.5%) was resistant to caspofungin, two (11.1%) were resistant to fluconazole, one (5.5%) was SDD to fluconazole, and one (5.5%) had a high MIC (2 µg/mL) to 5-fluorocytosine. Although the CLSI has not set any breakpoints for *C. dubliniensis*, the four isolates of this species presented low MIC values for all antifungals tested (Figure 2 and Table S1).

When comparing the geometric mean MIC among *Candida* species for each antifungal tested (Table S2), it was possible to establish that *C. parapsilosis* is less susceptible to all echinocandins than *C. tropicalis* and *C. glabrata* (*p* < 0.0001). On the contrary, *C. tropicalis* and *C. glabrata* were less susceptible to posaconazole, voriconazole, itraconazole, and fluconazole than *C. parapsilosis* (*p* < 0.01), with *C. glabrata* having higher MICs to all azoles than *C. tropicalis* (*p* < 0.01). *C. glabrata* was also less susceptible to amphotericin B than *C. parapsilosis*. The susceptibility of the studied isolates to 5-fluorocytosine did not differ depending on the species of *Candida* (Table S2).

### 3.4. ITS Sequencing Shows Different Levels of Intraspecies Genetic Diversity

In the studied isolates, the methods used in the hospital for the identification of *Candida* species agreed one hundred percent with the molecular method of ITS sequencing. Among the twenty-nine *C. parapsilosis* isolates studied, one SS and two haplotypes were identified in the sequences ranging from 517 to 552 bp, with most isolates (72.4%) grouping in a unique haplotype (D = 0.4138; Hd = 0.414; π = 0.0008) (Figure 2). Of the seventeen *C. tropicalis* isolates, six SSs and five haplotypes were identified in the sequences ranging from 483 to 550 bp, with most isolates (76.5%) grouping in a unique haplotype (D = 0.4265; Hd = 0.228; π = 0.0121). Among the 18 *C. glabrata* isolates, 12 SSs and 8 haplotypes were identified in the sequences ranging from 792 to 861 bp, with half the isolates (50%) grouping in a unique haplotype (D = 0.7516; Hd = 0.752; π = 0.0220). Only one haplotype and no SSs were identified among the four *C. dubliniensis* isolates, with sequences ranging from 548 to 566 bp.

## 4. Discussion

Considering their increasing global prevalence, antifungal resistance, and high risk of mortality, *C. glabrata*, *C. tropicalis*, and *C. parapsilosis* are currently ranked in the high-priority group of the fungal priority list recently issued by the WHO, together with *C. albicans* and *C. auris*, which are ranked in the critical group [19]. Therefore, studies like ours, addressing gaps in the local epidemiology of infections caused by these species of *Candida*, are important, not only to inform data on mortality, risk factors, and other characteristics of the affected patients, but also to better define trends in the incidence and antifungal susceptibility of these pathogens. In addition, this study emphasizes the need for the early recognition of *Candida* species, considering that, as they are globally occurring, these yeasts are among the most common pathogens causing BSIs [5]. A prompt and accurate diagnosis leads to correct antimicrobial therapy, and therefore improved patient outcomes.

In our centre, *C. albicans* is the major cause of invasive infection, causing almost 40% of all cases, with candidemia being the most common manifestation. Even though this prevalence is similar to that of other countries in the region and the world [9,10], it is of notice that, particularly in our hospital, *C. auris* accounts for a remarkable percentage of cases of invasive candidiasis. This is most likely because of its ability to cause nosocomial outbreaks and the difficulty in controlling it once it has been established in a hospital [16]. Notably, these local data coincide with the epidemiology of *C. auris* infections in the country, given that in Colombia this species has caused more than 1700 cases since its first report in 2016, and it is now the second most frequent species of *Candida* causing BSIs [16,30].

Similarly, the identification of *C. parapsilosis* and *C. tropicalis* in our centre as the first and second most frequent non-*albicans* and non-*auris Candida* species affecting critically ill patients agrees with the epidemiology of these species in Colombia and other countries in the region, including Argentina, Brazil, Chile, Ecuador, Honduras, and Venezuela [1,9,31,32]. However, the prevalence of *C. glabrata*, the third most frequent species in our centre, differs slightly with that of Argentina, Honduras, and Venezuela. In these three countries, *C. guilliermondii*, which was identified in one case in our hospital, equals or exceeds the number of cases of *C. glabrata* [9].

Interestingly, *C. glabrata*, which is the most common non-*albicans* species reported in most European countries, the USA, Canada, and Australia [1,12], was herein associated with a significantly higher mortality risk. Other studies have reported mortality rates for *C. glabrata* candidemia between 20 and 50%, and as high as 64%, particularly among untreated patients [19,33,34], which is similar to our rate of 62.5%. However, given that most patients who died of *C. glabrata* infections in our hospital received caspofungin treatment, it is possible that this high mortality might be associated with other underlying conditions or risk factors. In this study, *C. glabrata* was also characterized as having higher MIC values to azoles, with 11.1% and 5.5% of the isolates being resistant or SDD to fluconazole, respectively. Even though only one of our patients affected by *C. glabrata* received fluconazole, the identification of acquired resistance to this triazole, even without previous use, has long been reported not only in this species but also in *C. parapsilosis* [12,17,35]. In our study, 13.8% and 6.9% of the *C. parapsilosis* isolates were identified as resistant or SDD to fluconazole, respectively, which is concerning since ~60% of the patients affected by this species receive fluconazole as an initial therapy. While fluconazole is no longer the drug of choice for candidemia, this azole is still thought to be the best drug against *C. parapsilosis*, considering the trends toward better outcomes and the high MICs to echinocandins of this species, which also agree with our findings [12,36]. Fluconazole-resistant and SDD isolates were also identified in *C. tropicalis* (29.4%), which is a species that has generally lower rates of resistance to this azole than *C. glabrata* and *C. parapsilosis* [12,17,37]. This shows that, currently, the emergence of resistance threatens the efficacy of azoles not only against species with a high incidence of resistance to these antifungals, such as *C. glabrata*, but also against species that are becoming increasingly resistant, such as *C. parapsilosis* and *C. tropicalis*. Moreover, even though resistance to caspofungin was identified only in one isolate of *C. glabrata* (5.5%), some patients have been reported to have failed treatment with this echinocandin [12]. Overall, the identification of resistance and reduced susceptibility to one or more commonly used antifungal in non-*albicans* and non-*auris* species highlights the need to routinely carry out antifungal susceptibility testing as a key to adequate treatment, as well as the need to further investigate the mechanisms of antifungal resistance in emerging *Candida* species, which are largely unknown.

Apart from the displacement of *C. albicans* by *C. parapsilosis*, *C. auris*, *C. tropicalis*, and *C. glabrata*, which is occurring worldwide, the identification of less frequently recovered species, such as *C. dubliniensis* and *C. guilliermondii* in our hospital, reflects the rise in the diversity of human pathogens and the constant shift in the epidemiology of candidiasis and candidemia [11]. *C. dubliniensis*, which is recovered in less than 10% of the oral mucosa of healthy individuals, is usually associated with oropharyngeal infection, mainly in HIV/AIDS patients [38,39]. Interestingly, the patients from our centre affected by *C. dubliniensis* recovered from blood, BAL, and peritoneal lavage did not have evident immunosuppression, as they were HIV-seronegative. However, they had two or more underlying conditions, including diabetes mellitus, cancer, tuberculosis, and chronic obstructive pulmonary disease, some of which are additional comorbidities associated with a relatively high prevalence of *C. dubliniensis* [39,40,41]. Given that the spectrum of fungal species and at-risk hosts is widening, the proper identification of emerging species is therefore significant. Particularly, *C. dubliniensis* could be misidentified as *C. albicans* due to their phenotypic resemblance and close phylogenetic relationship [39,42].

The identification of low ITS genetic diversity among the *C. parapsilosis* isolates in this study agrees with other reports on its small intraspecific variation, suggesting that this species presents a clonal form of propagation in our hospital with a common reservoir of horizontal transmission, which must be identified as a preventive strategies [43,44]. Although *C. tropicalis* has been reported to have high ITS diversity, most isolates in our hospital were grouped into a main haplotype, which might suggest the clonal dispersal of this species [43]. In contrast, *C. glabrata* was found to have a higher genetic diversity, as previously reported, perhaps because this species is most frequently a human commensal involved in endogenously acquired infections [24,43]. Molecular studies based on ITS sequencing are hence important not only to confirm the identification given by conventional methods, but also to identify closely related and emerging species.

## 5. Conclusions

While this is a single-centre study with a relatively small sample size performed over a short period of time that does not provide insights related to generalizability across institutions, our study contributes to the epidemiology and depicts the clinical significance of non-*albicans* and non-*auris Candida* species, which is pertinent given that there is a major lack of data about many aspects of these pathogens, especially from middle- and low-income countries [19]. Furthermore, this study illustrates the importance of monitoring the emergence of acquired resistance, which might help to predict incoming challenges and might aid in guiding an appropriate empirical management of invasive infections. In addition to global studies, regional and local surveillance studies could better inform us on the annual incidence rates, distribution, and trends of the epidemiology of life-threatening fungal infections, such as invasive candidiasis, which in turn could improve diagnosis and generate preventive and therapeutic strategies. Knowing the clinical context of any underlying conditions and hospitalizations may be relevant to the initial clinical management of candidemia, treatment guidelines, and potential implications for patient outcomes.

## Figures and Tables

**Table 1:** Demographic and clinical characteristics of the studied patients.

Characteristic of Patients	Number (%)
Sex	
Male	31 (53.4)
Female	27 (46.6)
Underlying condition	
Antibiotic use	40 (69.0)
Bacterial co-infection	40 (69.0)
Cancer	21 (36.2)
High blood pressure	21 (36.2)
Antihypertensives use	17 (29.3)
Diabetes mellitus	17 (29.3)
Chronic kidney disease	10 (17.2)
COPD	9 (15.5)
Coronary artery disease	9 (15.5)
CVA	9 (15.5)
HSCT	8 (13.8)
Other	50 (86.2)
Diagnosis	
Candidemia	29 (50.0)
Invasive candidiasis	21 (36.2)
Urinary tract infection	8 (13.8)
Treatment ^1^	
Fluconazole	21 (36.2)
Caspofungin	19 (32.8)
Amphotericin B	5 (8.6)
Unknown	3 (5.2)
None	19 (32.8)
Outcome	
Alive	31 (53.4)
Dead	27 (46.6)
	**Mean (range)**
Age (years)	63.1 (18 days—93)
Length of hospital stay (days)	36.1 (0–114)

^1^ Usage of the antifungal alone or in combination. COPD: chronic obstructive pulmonary disease. CVA: cerebrovascular accident. HSCT: hematopoietic stem cell transplant.

**Table 2:** Multivariate analysis of characteristics associated with the outcome of patients.

		Survived *n* = 31	Died *n* = 27	OR (95% CI)	*p*-Value
**Sex**	Male	16 (27.6%)	15 (25.9%)	0.853 [0.41–1.77]	0.812
	Female	15 (25.9%)	12 (20.7%)	Reference	
**Age**	Mean in years (SD)	61.9 (±19.8)	65.6 (±17.9)	-	0.365
	Risk for each 1-year increase			1.01 [0.99–1.03]	0.2996
**Length of hospital stay**	Mean in days (SD)	35.4 (±27.2)	36.7 (±24.6)	-	0.66
	Risk for each 1-day increase			0.998 [0.984–1.01]	0.796
**Diagnosis**	Candidemia	14 (24.1%)	15 (25.9%)	1.07 [0.354–3.24]	0.903
	Invasive candidiasis	13 (22.4%)	8 (13.8%)	4.36 [0.193–1.97]	0.412
	Urinary tract infection	4 (6.9%)	4 (6.9%)	Reference	
**Hospitalization**	ICU	19 (32.8%)	22 (37.9%)	0.36 [0.153–0.847]	**0.0193**
	General ward	12 (20.7%)	5 (8.6%)	Reference	
**Species**	*C. parapsilosis*	12 (20.7%)	10 (17.2%)	Reference	
	*C. tropicalis*	9 (15.5%)	7 (12.1%)	0.812 [0.298–2.21]	0.683
	*C. glabrata*	6 (10.3%)	10 (17.2%)	0.304 [0.104–0.894]	**0.0305**

CI: confidence interval; ICU: intensive care unit; SD: standard deviation; OR: odds ratio.

**Table 3:** Univariate analysis of characteristics associated with the outcome of patients.

		Survived *n* = 31	Died *n* = 27	OR (95% CI)	*p*-Value
**Underlying condition**	Antibiotic use	18	22	3.18 [1.36–7.45]	**0.012**
	Co-infection	22	18	0.818 [0.372–1.8]	0.618
	Cancer	9	12	1.87 [0.864–4.03]	0.112
	High blood pressure	12	9	0.792 [0.369–1.7]	0.548
	Antihypertensives use	7	10	2.02 [0.895–4.54]	0.0904
	Diabetes mellitus	12	5	0.36 [0.153–0.847]	**0.0193**
	Chronic kidney disease	4	6	1.93 [0.723–5.15]	0.19
	Other	20	20	1.57 [0.706–3.5]	0.269
**Diagnosis**	Candidemia	14	15	0.531 [0.233–1.21]	0.13
	Invasive candidiasis	13	8	1.99 [0.825–4.82]	0.125
	Urinary tract infection	4	4	1.07 [0.302–3.76]	0.92
**Species**	*C. parapsilosis*	12	10	1.82 [0.787–4.21]	0.162
	*C. tropicalis*	9	7	1.26 [0.518–3.05]	0.613
	*C. glabrata*	6	10	0.337 [0.128–0.89]	**0.0282**

CI: confidence interval; OR: odds ratio.

## Data Availability

All data generated or analysed during this study are included in this published article and its Supplementary Information files. The DNA sequences obtained, per isolate, were deposited in GenBank under the following accession numbers: OR859770 to OR859837.

## Supplementary Materials

The following supporting information can be downloaded at: https://www.mdpi.com/article/10.3390/jof10050326/s1, Table S1: Distribution, per species and per antifungal, of the minimum inhibitory concentration (MIC) values of the studied *Candida* isolates (n = 68); Table S2: Comparison of minimum inhibitory concentration values per antifungal between *Candida* species.
