# Supplementary material for: Candida Non-albicans and Non-auris Causing Invasive Candidiasis in a Fourth-Level Hospital in Colombia: Epidemiology, Antifungal Susceptibility, and Genetic Diversity
Source: J Fungi (Basel). 2024 Apr 30;10(5):326. doi: 10.3390/jof10050326 (PMC11122357; doi:10.3390/jof10050326)
Supplement: Supplementary file 1 [file jof-10-00326-s001.zip › jof-2990158-supplementary.pdf]

Supplementary material

Table S1. Distribution, per species and per antifungal, of the minimum inhibitory concentration (MIC) values of the studied *Candida* isolates (n=68)

				**No. of isolates at MIC value (µg/ml)[1]**															
**Antifungal[2]**	**Species**	**n**	**GM[3]**	**0,008**	**0,016**	**0,03**	**0,06**	**0,12**	**0,25**	**0,5**	**1**	**2**	**4**	**8**	**16**	**32**	**64**	**128**	**256**
AND	*C. dubliniensis*	4	0.04419		2	-	-	2											
	C. glabrata	18	0.03937			14	2	2											
	C. parapsilosis	29	0.5245					2	5	15	3	4							
	C. tropicalis	17	0.02765		8	5	3	1											
MF	*C. dubliniensis*	4	0.0221	1	1	1	1												
	C. glabrata	18	0.01688	2	12	4													
	C. parapsilosis	29	0.8064						3	8	13	5							
	C. tropicalis	17	0.02655		5	11	1												
CAS	*C. dubliniensis*	4	0.05256		1	1		2											
	C. glabrata	18	0.08505			1	10	6		**1**									
	C. parapsilosis	29	0.2886					5	14	9	1								
	C. tropicalis	17	0.02079	1	8	8													
FC	*C. dubliniensis*	4	0.06250				4												
	C. glabrata	18	0.07577				17	-	-	-	-	1							
	C. parapsilosis	29	0.07043				24	5											
	C. tropicalis	17	0.2124				14	-	-	-	-	-	-	-	-	-	**3**		
PZ	*C. dubliniensis*	4	0.04419			2	2												
	C. glabrata	18	0.9259					1	1	1	13	1	-	1					
	C. parapsilosis	29	0.03051		10	11	7	1											
	C. tropicalis	17	0.2212					6	8	3									
VOR	*C. dubliniensis*	4	0.01314	1	3														
	C. glabrata	18	0.3819				1	2	6	6	1	1	1						
	C. parapsilosis	29	0.03278	5	9	5	5	1	2	2									
	C. tropicalis	17	0.1879				4	6	4	2	-	-	-	**1**					
IZ	*C. dubliniensis*	4	0.04419		2	-	-	2											
	C. glabrata	18	0.6062					1	3	9	3	1	-	-	1				
	C. parapsilosis	29	0.06401			9	10	10											
	C. tropicalis	17	0.2500					1	15	1									
FZ	*C. dubliniensis*	4	0.500							4									

	C. glabrata	18	9.699					2	3	7	3	1	**1**	**1**	
	C. parapsilosis	29	1.240		5	3	12	3	2	**1**	**3**				
	C. tropicalis	17	2.771				4	8	3	**1**	-	-	-	-	**1**
AMB	*C. dubliniensis*	4	0.2973		3	1									
	C. glabrata	18	0.7349	1	-	5	12								
	C. parapsilosis	29	0.500	1	3	20	5								
	C. tropicalis	17	0.5886	1	-	10	6								

[1]The modal MIC for each distribution is underlined. Resistant isolates are in bold.

[2]AMB: Amphotericin-B; AND: anidulafungin; CAS: caspofungin; FC: 5-fluorocytosine; FZ: Fluconazole; IZ: Itraconazole; MF: Micafungin; PZ: Posaconazole, VOR: Voriconazole.

[3]GM: Geometric mean in μg/ml.

Table S2. Comparison of minimum inhibitory concentration values, per antifungal, between *Candida* species. Geometric mean MICs in μg/ml are compared and *p*-values for statistical significance are shown in parenthesis.

	C. parapsilosis vs. C. glabrata	***C. parapsilosis vs. C. tropicalis***	***C. glabrata vs. C. tropicalis***
AND	0,5245 vs. 0,03937 (<0,0001)	0,5245 vs. 0,02765 (<0,0001)	0,03937 vs. 0,02765 (0,0372)
MF	0,8064 vs. 0,01688 (<0,0001)	0,8064 vs. 0,02655 (<0,0001)	0,01688 vs. 0,02655 (0,0032)
CAS	0,2886 vs. 0,08505 (<0,0001)	0,2886 vs. 0,02079 (<0,0001)	0,08505 vs. 0,02079 (<0,0001)
FC	0,07043 vs. 0,07577 (0,3839)[ns]	0,07043 vs. 0,2174 (0,6960)[ns]	0,07577 vs. 0,2174 (0,1623)[ns]
PZ	0,03051 vs. 0,9259 (<0,0001)	0,03051 vs. 0,2212 (<0,0001)	0,9259 vs. 0,2212 (<0,0001)
VOR	0,03278 vs. 0,3819 (<0,0001)	0,03278 vs. 0,1879 (<0,0001)	0,3819 vs. 0,1879 (0,0126)
IZ	0,06401 vs. 0,6062 (<0,0001)	0,06401 vs. 0,2500 (<0,0001)	0,6062 vs. 0,2500 (0,0004)
FZ	1,240 vs. 9,699 (<0,0001)	1,240 vs. 2,771 (0,0104)	9,699 vs. 2,771 (0,0001)
AMB	0,50000 vs. 0,7349 (0,0011)	0,50000 vs. 0,5886 (0,1673)[ns]	0,7349 vs. 0,5886 (0,1220)[ns]

AMB: Amphotericin-B; AND: anidulafungin; CAS: caspofungin; FC: 5-Fluorocytosine; FZ: Fluconazole; IZ: Itraconazole; MF: Micafungin; PZ: Posaconazole, VOR: Voriconazole.
ns: not significant
